# Ethical values and principles to guide the fair allocation of resources in response to a pandemic: a rapid systematic review

**DOI:** 10.1186/s12910-022-00806-8

**Published:** 2022-07-07

**Authors:** Lydia O’Sullivan, Edelweiss Aldasoro, Áine O’Brien, Maeve Nolan, Cliona McGovern, Áine Carroll

**Affiliations:** 1 School of Medicine, University College Dublin, Dublin 4, Ireland; 2 Health Research Board-Trials Methodology Research Network, National University of Ireland Galway, Galway, Ireland; 3 International Foundation for Integrated Care, Annexe Offices, Linton Road, Oxford, OX2 6UD England; 4 Limerick, Ireland; 5 National Rehabilitation Hospital, Dun Laoghaire, Dublin, A96 E2H2 Ireland; 6 International Foundation for Integrated Care and the National Rehabilitation Hospital, Dun Laoghaire, Dublin, Ireland

**Keywords:** COVID-19, SARS-CoV-2, Pandemic, Emergencies, Ethics, Ethical principles, Ethical frameworks, Equity, Resource allocation, Healthcare resources

## Abstract

**Background:**

The coronavirus 2019 pandemic placed unprecedented pressures on healthcare services and magnified ethical dilemmas related to how resources should be allocated. These resources include, among others, personal protective equipment, personnel, life-saving equipment, and vaccines. Decision-makers have therefore sought ethical decision-making tools so that resources are distributed both swiftly and equitably. To support the development of such a decision-making tool, a systematic review of the literature on relevant ethical values and principles was undertaken. The aim of this review was to identify ethical values and principles in the literature which relate to the equitable allocation of resources in response to an acute public health threat, such as a pandemic.

**Methods:**

A rapid systematic review was conducted using MEDLINE, EMBASE, Google Scholar, LitCOVID and relevant reference lists. The time period of the search was January 2000 to 6th April 2020, and the search was restricted to human studies. January 2000 was selected as a start date as the aim was to capture ethical values and principles within acute public health threat situations. No restrictions were made with regard to language. Ethical values and principles were extracted and examined thematically.

**Results:**

A total of 1,618 articles were identified. After screening and application of eligibility criteria, 169 papers were included in the thematic synthesis. The most commonly mentioned ethical values and principles were: Equity, reciprocity, transparency, justice, duty to care, liberty, utility, stewardship, trust and proportionality. In some cases, ethical principles were conflicting, for example, Protection of the Public from Harm and Liberty.

**Conclusions:**

Allocation of resources in response to acute public health threats is challenging and must be simultaneously guided by many ethical principles and values. Ethical decision-making strategies and the prioritisation of different principles and values needs to be discussed with the public in order to prepare for future public health threats. An evidence-based tool to guide decision-makers in making difficult decisions is required. The equitable allocation of resources in response to an acute public health threat is challenging, and many ethical principles may be applied simultaneously. An evidence-based tool to support difficult decisions would be helpful to guide decision-makers.

**Supplementary Information:**

The online version contains supplementary material available at 10.1186/s12910-022-00806-8.

## Background

The Severe Acute Respiratory Syndrome coronavirus 2 pandemic, otherwise known as the coronavirus 2019 (COVID-19) pandemic, quickly overwhelmed the most sophisticated of healthcare systems, placing unprecedented pressure on healthcare services. The pandemic has also magnified many ethical issues related to the provision of appropriate standards of care, privacy and confidentiality, informed consent, community engagement, benefit-sharing and resource allocation [1].

Although such a pandemic has long been anticipated, with published recommendations for countries to use in their preparations [2, 3], many countries have struggled to allocate resources and apply control measures. As Thomas and colleagues noted, considering the ethics of a situation requires ethical reflection and discussion, skills that require preparation and practice [4].

In Ireland, as in many neighbouring European countries, the pandemic has forced a shift from person-centred healthcare provision to practices primarily guided by considerations on the well-being of the population as a whole [5]. Due to the overwhelming nature of the pandemic, with demand outstripping capacity in many countries it has been challenging to adhere to a ‘duty of care’ model and respond in an equitable, reasonable, and proportionate way. The published experience of many countries has shown that pandemics can be catastrophic on healthcare systems, decimating resources (i.e. protective equipment), and resulting in a shortage of personnel and life-saving equipment [6–11]. The available literature demonstrates that when faced with an increasing number of people requiring acute care, ethical decisions are required on the allocation of resources. Unique and challenging ethical issues have been raised as a direct result of COVID-19. These include prioritising access to healthcare resources, obligations of frontline workers considering the risk to their own and their families’ health, and the implementation of measures to reduce the spread of the infection while protecting the rights of the individual.

In this context, decision-makers are looking for ethical decision-making tools providing key knowledge-sharing opportunities and complimenting decisions on care provision and delivery, as swiftly—but proportionally and fairly, as possible. Decision-making tools are required, among other reasons, to promote transparency and maintain accountability with policy makers, to ensure collective justice and to encourage engagement with healthcare providers working on the front lines.

After the World Health Organisation (WHO) pronounced the COVID-19 outbreak a pandemic in March 2020, and after the first cases of COVID-19 were reported in Ireland (in late February 2019), the National Rehabilitation Hospital (NRH) in Ireland, a complex specialist rehabilitation facility, established the COVID-response committee. The NRH provides complex specialist rehabilitation services to patients who, as a result of an accident, illness or Injury, have acquired a physical or cognitive disability and would therefore be considered vulnerable [12]. Decisions were rapidly taken to limit risks to staff and patients. It was recognised that such decisions had ethical dimensions, and in the absence of national guidance at that time, the matter was escalated to the Hospital Ethics Committee for consideration. The committee recognised that to support hospital management in its decision-making, they needed to be evidence-informed and requested that a rapid review of the literature be completed and presented to the committee. A research team was swiftly convened to conduct a rapid systematic review. The aim of the rapid systematic review was to identify ethical values and principles which related to the equitable allocation of resources in the context of an acute public health threat [13], such as a pandemic. The results of this rapid systematic review were used to support the development of an evidence-based ethical framework to provide guidance on the ethical allocation of resources. It is expected that such a framework would have applicability to a wide range of national and international healthcare settings.

## Methods

### Scope of the review

A rapid systematic review methodology was selected given the time-sensitive nature of this project. As described by Tricco and colleagues, ‘*Rapid reviews are a form of knowledge synthesis in which the components of the systematic review process are simplified or omitted to produce information in a timely manner*’ [14]. Rapid reviews have emerged as a streamlined approach to knowledge synthesis, usually to inform urgent decisions faced by decision-makers in a healthcare setting [15, 16]. Although the review team were required to respond to the time-sensitive needs of the ethics committee, they simultaneously had to ensure that the scientific imperative of methodological rigour was satisfied.

The research team consisted of a core team of three researchers who performed the database searching, screening and data extraction, and a broader steering group including a medical ethicist, an academic medical consultant and a clinical psychologist. This team set and refined the review question, eligibility criteria, and the outcomes of interest.

The review protocol was developed in line with the PICO evidence-based approach (Problem, Intervention, Comparator, Outcome) which was used to frame the research question [17] as follows –

- *Problem* In a context of acute resource limitations in healthcare system, how should limited resources be rationed or allocated fairly in the healthcare setting?
- *Intervention* ethical values or principles to guide allocation of resources
- *Comparator* not applicable for this review
- *Outcome* maximise the protection of a person’s rights to healthcare, minimise the risk for treatment withdrawal based on unethical reasoning, and support for practitioners, administrators and managers making difficult decisions regarding resource allocation.

The protocol was published on the Open Science Framework and is available at https://osf.io/krgsn/

The review was reported in accordance with the Preferred Reporting Items for Systematic Reviews and Meta-Analyses (PRISMA) guidance and checklist [18]—see Additional File 1.

### Search strategy

The search strategy was informed and refined with advice from an information specialist (health sciences liaison librarian). The search is described with reference to the PRISMA-S checklist [19]—see Additional File 2.

A comprehensive literature review search of multiple bibliographic databases including MEDLINE, EMBASE and Google Scholar was conducted. Google Scholar was selected as an effective tool to identify grey literature [20, 21]. A search of references lists of relevant systematic reviews, government and non-governmental organisation reports, opinion pieces, included articles and other relevant grey literature, including LitCOVID was also undertaken. National and International Ethical Frameworks already familiar to the authors were also included. References in identified articles were also reviewed (backwards citation screening).

The search terms used for the MEDLINE search within the title or abstract are shown in Table 1.

**Table 1:** Search terms used in MEDLINE

Search string	Key words
1	(“Coronavirus” OR “COVID19” OR “epidemic” OR “outbreak” OR “pandemic” OR “humanitarian emergency” OR “catastrophes” OR “disaster”)
2	(“Resources” OR “Resource Allocation” OR “Rationing” OR “Shortage” OR “Personal Protective Equipment” “Ventilator” OR “Triage” OR “Withholding”)
3	(“Ethics” OR “Morality” OR “Ethical framework” OR “Health equity” OR “Decision making”)
4	1 AND 2 AND 3
5	Limit Jan 2000 to 6^th^ April 2020

A list of the EMTREE (EMBASE) search terms can be found in Additional File 3. No limits with regard to language were applied.

For MEDLINE and EMBASE, the following limits were applied:

- Human studies
- Time period January 2000 to 6th April 2020

Within Google Scholar, the search was performed in incognito mode—this ensures that any previous searches will not influence Google’s algorithm when searching for new material/ The first 200 entries were included, as recommended by Haddaway and colleagues [20].

All identified papers were exported to Zotero, and duplicates excluded. All remaining papers were imported to Rayaan for review [22]. Two members of the team (LOS and AOB) independently and blindly screened Titles and Abstracts of all papers in accordance with the Inclusion and Exclusion Criteria detailed below. Additionally, the scope of this rapid review was limited to public health threats, such as pandemics. For this reason, disasters such as plane crashes or hurricane aftermath, where the triage of casualties would be required, were excluded.

### Study selection

#### Title/abstract screening

Articles were included if they met the following Inclusion/Exclusion Criteria.

Inclusion Criteria—selected papers including at least two of the following three concepts:

- Acute Resource Limitation or similar
- Rationing / Allocation / Decision-making or similar
- Ethical perspective

Exclusion Criteria—papers with no abstract or with a focus on the following were excluded:

- Accident and Emergency Services
- In-flight emergencies
- Clinical research taking place during humanitarian emergencies
- Ethics in clinical research
- Communication strategies
- Informed consent
- Other clinical emergencies which are not outbreaks/disasters/pandemics

Once Title/Abstract screening was complete, a third member of the research team (EA) resolved any conflicts.

Authors of individual papers were not contacted to collect additional information or to request the full text of inaccessible papers, due to time constraints.

#### Full text screening

All three team members (EA, LOS, AOB) each reviewed a portion of the full texts. Papers were excluded if:

- The full text was not available
- They did not contain principles or values relevant to an ethical framework which could be used to support decision-making about the allocation of resources
- The topic related solely to triage procedures in pandemics/disasters e.g., operational medical or nursing triage procedures
- The topic related to legal aspects, rather than ethical ones
- They contained only a clinical case study/case studies i.e., summary of a patient or patients’ clinical condition and outcome
- They only gave a brief summary of the main ethical approaches, with no further details
- The topic related only to pandemic planning

### Data extraction and analysis

Data extraction was completed by all three reviewers using a customised data extraction form in Microsoft Excel, all three checked for correctness and completeness of extracted data, and one researcher synthesised all data extractions. The following data items were extracted:

- Lead author
- Year of publication
- Ethical values or principles relevant to an ethical framework
- Synonyms of the named ethical value or principle
- Related ethical values or principles
- Example(s) of a scenario where the ethical values or principles apply

The aim of this rapid systematic review was to identify ethical values and principles, rather than quantitatively assess healthcare interventions or to assess the methodological quality of clinical trials. Therefore, the articles were assessed, and the extracted themes were synthesised, and no risk of bias assessment was performed. Emphasis was placed on high-quality literature and also key publications identified by the key stakeholders—these included peer-reviewed literature, government reports or publications produced by reputable organisations. Due to differences in nomenclature used in the literature, both the terms ‘ethical value’ and ‘ethical principle’ are used to describe the findings.

## Results

### Article inclusion

**Fig. 1:**
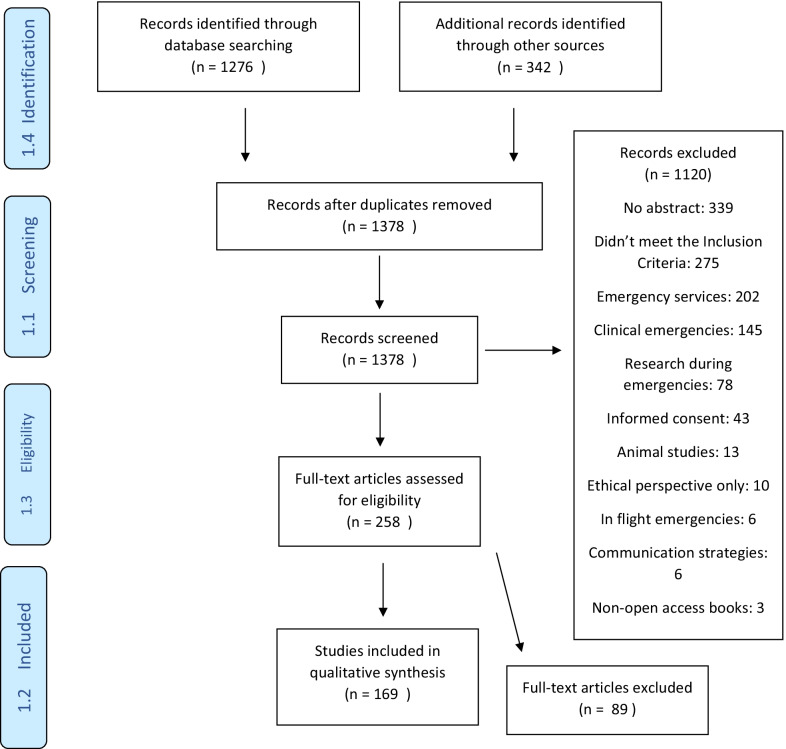
PRISMA flow diagram of the evidence identified by this rapid review

A PRISMA flow diagram of the evidence identified by this rapid review is shown in Fig. 1.

A total of 1276 articles were obtained from the electronic search of international databases, and an additional 342 articles were identified through other sources. After screening and application of eligibility criteria, 169 papers were included in the thematic synthesis.

### Characteristics of included articles

The main types of articles included in the full text review were policy papers, e.g. [23–25], discussion papers, e.g. [26, 27], ethical debates, e.g. [28–30] or case studies from previous disaster situations, e.g. [31–33]. Several articles were publications from national or local governments, such as departments of health [5, 34–40].

### Ethical values and principles

31 ethical values and principles were identified from the 169 full text articles. Table 2 summarises the ethical principles and values identified. For brevity, only the most recent references are included, but the full list of references is included in Additional File 4. Equity, reciprocity, transparency, justice, duty to care, liberty, utility, stewardship, trust and proportionality were the most common values and principles identified. These values and principles were applied to a wide range of scenarios, including terrorism [41, 42], vaccination distribution [43–45] and quarantine measures such as lockdowns [24, 46–48].

**Table 2:** Ethical values and principles extracted from included studies

1	Equity [43]	Fairness [14]	49	British Medical Association, 2020 [90]
		Equality [4]		Chisholm, 2020 [91]
		Antidiscrimination, Non-discriminatory [3]		
		Fair distribution [1]		
		Legitimacy [1]		
		Justice as fairness [1]		
2	Reciprocity [24]	Mutual exchange [1]	24	Berlinger, 2020 [92]
		Society and employers should support and protect		British Medical Association, 2020 [90]
		those who take on increased burden and risk [4]		
		Support for those enduring a disproportionate burden during crisis and address/minimise burden [1]		
		Obligations to healthcare workers [1]		
		Justice-orientated reciprocity [1]		
3	Transparency [19]	Openness and public accessibility [2]	21	British Medical Association, 2020 [90]
		Communication [2]		Scottish Government, 2020 [93]
		Publicly defensible [1]		
		Justification [1]		
		Veracity [1]		
4	(Social) (Distributive) Justice [17]	Justice as fairness [1]	18	White, 2020 [94]
				Centers for Disease Control and Prevention, 2019 [95]
5	Duty to (provide) care [14]	Professional obligation of non-abandonment [1]	18	Department of Health Ireland, 2020 [96]
		Professional duty to respond [1]		Gostin, 2020 [97]
		Professional responsibility 1]		
		Duty to treat [1]		
		The obligation of healthcare workers to serve under stressful and risky conditions [1]		
		Professional integrity [1]		
6	Individual Liberty [10]	Liberty [4]	18	Gostin, 2020 [97]
		Least restrictive [3]		White 2020 [94]
		Autonomy [2]		
		Constraints on / restrictions of liberty [3]		
		Individual autonomy [1]		
		Equal liberty and human rights [1]		
		Patient autonomy [1]		
		Patient liberty [1]		
		Choice, Free-will, Self-determination [1]		
7	Utility [9]	Efficiency [1]	10	Emanuel, 2020 [53]
		Effectiveness [1]		Ram-Tiktin [31], 2017
		Greatest good for the greatest number [1]		
		Utilitarian value [1]		
8	Stewardship [11]	Governance [1]	13	Ryus, 2018 [12]
		Duty to steward resources [1]		Ra-Tiktin, 2017 [31]
9	Trust [9]	Informed and trusted communication [1]	12	Gostin, 2020 [97]
		Fidelity [1]		Eyal, 2012 [98]
		Honouring Patients’ Trust [1]		
10	Proportionality [9]	Fair procedures [1]	10	Alberta Government, 2016 [99]
				Mariaselvam, 2016 [100]
11	Accountability [8]		8	Centers for Disease Control and Prevention, 2019 [95]
				Ryus, 2018 [101]
12	Privacy [5]		5	Department of Health, Ireland, 2020 [96]
				Barnett, 2009 [102]
13	Beneficence [4]	Avoid harm, harm reduction, minimising harm [4]	9	Gostin, 2020 [97]
		Nonmaleficence [1]		British Medical Association, 2020 [90]
14	Protection of the Public from Harm [4]	Good preparedness [1]	6	Gostin, 2020 [97]
		Protection of individuals at highest risk, meeting societal needs, and promoting social justice [1]		Mariaselvam, 2016 [100]
		Ensuring that benefits of relief and rescue activities reach the affected [1]		
15	Autonomy [4]		4	Kukora 2016 [103]
				Kirby, 2010 [104]
16	Solidarity [3]	Mutual responsibility [1]	4	Johns Hopkins Berman Institute of Bioethics, 2020 [105]
				Silva, 2012 [67]
17	Working together [3]		3	British Medical Association, 2020 [90]
				Scottish Government, 2020 [93]
18	Community participation [2]	Community resilience and empowerment [1]	4	Centers for Disease Control and Prevention, 2019 [95]
		Obligations to community [1]		Mariaselvam, 2016 [100]
19	Responsiveness [2]	Responsiveness to local values [1]	3	Mariaselvam, 2016 [100]
				Trotter, 2010 [106]
20	Consistency [2]		2	Ryus, 2018 [101]
				Hick, 2012 [107]
21	Duty to Plan [1]	Flexibility and adaptability [1]	2	British Medical Association, 2020 [90]
				Ryus, 2018 [101]
22	Evidence [1]		1	Barnett, 2009 [102]
23	Others: related to Social-Community	Respect [2]		(*)
		Social cohesiveness and collaboration [1]		
		Responsive civic response [1]		
		Dignity [1]		
		Compassion [1]		
24	Others: related to decision-making processes	Reasonableness [1]		(*)
		Inclusiveness [1]		
		Sustainability (sustainable action and sustainable outcomes) [1]		
		Relevance [1]		

(*) The most recent references presented in the table. For full reference list of each entry see Additional File 4

It was noted that while some ethical principles were complimentary, e.g., solidarity, social cohesiveness and collaboration, others were potentially in conflict, e.g., liberty/autonomy, and protection of the public from harm. Another example of conflicting ethical principles related to the duty to provide care and reciprocity as healthcare and other frontline workers can be exposed to additional risks while performing their duties in disaster situations.

While there was broad agreement within the included studies regarding the importance of applying the principles of fairness, trust, equity etc., there was some discordance regarding the application of a utilitarian versus an egalitarian perspective [49]. While most authors did not espouse the utilitarian approach, a small number felt that this principle should apply in disaster situations in deciding how resources should be distributed [31, 50–54]. Others felt that utilitarianism should be combined with the principle of fairness, rather than applied in isolation [49, 55].

Several authors noted that the principle of reciprocity might apply to key workers, e.g., healthcare or frontline workers who are at the greatest risk and whose role is crucial to resolving the disaster situation [56–60]. Several studies referred to the ethical values and dilemmas for healthcare professionals arising from their willingness to work in situations of personal danger [61–64].

Several authors emphasised the importance of taking a pre-planned, objective, structured approach when allocating resources, to ensure fairness [5, 65, 66]. Other authors emphasised the importance of public consultation regarding ethical values in a disaster situation, in order to maintain public trust [32, 67–76], bearing in mind that ethical values will vary depending on the local culture [77].

Some authors specifically noted the importance of considering marginalised populations who may have difficulties accessing healthcare [78–80]. Similarly, several authors noted the importance of social justice, for example, with regard to the fair distribution of vaccines globally [44, 45].

## Discussion

### Summary of key findings

The most frequently cited ethical values and principles included equity, reciprocity, transparency, justice, duty to care, liberty, utility, stewardship, trust, and proportionality. It was also noted that in some cases, there may be a conflict between values and principles—e.g., between liberty/autonomy and protection of the public from harm. The importance of a pre-planned, structured approach, informed by public consultation was evident, as well as the inclusion of marginalised populations and countries with fewer resources.

### Application of the principle of equity and social justice

The COVID-19 pandemic has exposed a range of social inequalities from crowded living conditions, barriers to accessing healthcare and COVID-19 testing, lower-paid workers having higher rates of exposure, higher rates of transmission of infection for those using public transport, or with public-facing jobs [81, 82]. Currently, developed countries are purchasing stocks of vaccines and have begun vaccination programmes, while developing countries are likely to fall further behind. Even within developed countries, diverse groups are staking their claim to receive the vaccine as a priority and difficult decisions have to be made regarding prioritisation [83]. This demonstrates the importance of employing decision-making tools firmly based on ethical principles and values. It is also evident that lower-income countries, with lesser resources, may face even more difficult decisions with regard to the allocation of resources. It is also important to note that ethical values and principles will vary from culture to culture, emphasising the importance of local engagement.

### Considerations for future research

The importance of incorporating ethical decision-making into pandemic planning was highlighted during the previous Severe Acute Respiratory Syndrome (SARS) outbreak [84]. During this outbreak, ethical issues were predominantly raised by public health, governments and healthcare workers as opposed to logistical and scientific issues [85, 86]. Furthermore, the absence of clear ethical guidelines during the SARS pandemic resulted in the loss of public trust, low morale amongst healthcare workers, confusion regarding roles and responsibilities, stigmatisation of vulnerable individuals, communities, misinformation and public fear [87].

Ethics contributes minimally to the understanding and mechanism of COVID-19 virus transmission. However, it significantly informs the decision-making process in relation to best clinical practice, the level of harm the public are expected and prepared to accept, how the burden of negative outcomes for specific populations should be addressed and if investment in additional resources is required. It is beyond the scope of this rapid review to discuss the application of each of these ethical principles and values; co-production with relevant stakeholders is needed within individual contexts. However, this rapid systematic review was used to support the development of an evidence-based ethical framework to provide guidance on an ethical process for decision-making on substantive clinical issues, incorporating evidence-based ethical values and thereby potentially mitigating unintended and avoidable collateral damage from COVID-19.

### Strengths and limitations

The main advantage of using the rapid systematic review approach is the speed with which evidence can be synthesised. This can provide decision-makers with evidence to inform action, which is particularly important in a pandemic or disaster situation [88]. By incorporating two of the commonly used databases (MEDLINE and EMBASE), in addition to Google Scholar and assessment of grey literature, without language restrictions, a comprehensive review of the literature was completed. However, streamlining the systematic review process, e.g., only a single researcher performing each data extraction, may have introduced some level of bias [89]. There are also limitations associated with using Google Scholar, such as difficulties with reproducibility. As with any rapid review, a balance was sought between rigour and speed. It is also acknowledged that there was also a certain level of linguistic subjectivity associated with the categorization of ethical principles and values identified in this review, and that this a limitation.

## Conclusions

Allocation of resources during a pandemic is a complex task, fraught with ethical dilemmas. It is crucial that decision-making in a pandemic is based on the principles of social justice regarding the allocation of resources. This systematic review identifies widely used and valued ethical principles which could be used to inform an ethical framework to support difficult decisions in a time of crisis.

## Supplementary Information

**Additional file 1.** PRISMA checklist.

**Additional file 2.** PRISMA-S checklist.

**Additional file 3.** EMBASE search terms.

**Additional file 4.** Full reference list of included papers.

## Data Availability

The datasets used and/or analysed during the current study are available from the corresponding author on reasonable request.

## Abbreviations

**COVID-19**: Coronavirus disease 2019
**EMBASE**: Excerpta Medica database
**MEDLINE**: Medical literature analysis and retrieval system online
**NRH**: National Rehabilitation Hospital
**PICO**: Population; intervention; comparator; outcome
**PRISMA**: Preferred reporting items for systematic reviews and meta-analyses
**SARS**: Severe acute respiratory syndrome
**SARS-CoV-2**: Severe acute respiratory syndrome coronavirus 2
**WHO**: World Health Organisation
