# Supplementary material for: Ethical values and principles to guide the fair allocation of resources in response to a pandemic: a rapid systematic review
Source: BMC Med Ethics. 2022 Jul 7;23:70. doi: 10.1186/s12910-022-00806-8 (PMC9261249; doi:10.1186/s12910-022-00806-8)
Supplement: Supplementary file 3 — Additional file 3. EMBASE search terms. [file 12910_2022_806_MOESM3_ESM.pdf]

EMBASE: Full list of terms

1. ("coronavirus disease 2019' OR "2019 novel coronavirus disease" OR "2019 novel coronavirus infection" OR "2019-nCoV disease" OR "2019-nCoV infection" OR "COVID" OR "COVID 19" OR "COVID 2019" OR "COVID-19" OR "COVID19" OR "nCoV 2019 disease" OR "nCoV 2019 infection" OR "novel coronavirus 2019 disease" OR "novel coronavirus 2019 infection" OR "novel coronavirus disease 2019" OR "novel coronavirus infection 2019" OR "SARS coronavirus 2 infection" OR "SARS-CoV-2 disease" OR "SARS-CoV-2 infection" OR "SARS-CoV2 disease" OR "SARS-CoV2 infection" OR "SARSCoV2 disease" OR "SARSCoV2 infection" OR "Wuhan coronavirus disease" OR "Wuhan coronavirus infection" OR "epidemic" OR "disease outbreaks" OR "epidemics" OR "pandemic" OR "pandemics" OR "humanitarian crisis" OR "humanitarian disaster" OR "disaster")

2. ("low resources" OR "scarce resources" OR "resource allocation" OR "fair allocation" OR "rationing" OR "shortage" OR "ventilator" OR "personal protective equipment" OR "PPE" OR "protective equipment" OR "triage" OR "treatment withdrawal" OR "withholding treatment")

3. ("ethics" OR "medical ethics" OR "medical morale" OR "ethical framework" OR "health equity" OR "ethical decision-making")
