# Supplementary material for: Ethical values and principles to guide the fair allocation of resources in response to a pandemic: a rapid systematic review
Source: BMC Med Ethics. 2022 Jul 7;23:70. doi: 10.1186/s12910-022-00806-8 (PMC9261249; doi:10.1186/s12910-022-00806-8)
Supplement: Supplementary file 4 — Additional file 4. Full reference list of included papers. [file 12910_2022_806_MOESM4_ESM.pdf]

APPENDIX D: Full reference list of included papers.

	Value or principle	Similar concepts / Synonyms	Comments and related concepts	No. of sources the value or the synonym are mentioned
1	Equity (1-43)	- Fairness(4, 12-13, 19, 29, 32, 35, 42, 44-49)<br>- Equality (3, 11, 32, 50)<br>- Antidiscrimination, Non-discriminatory (27, 50-51)<br>- Fair distribution (10)<br>- Legitimacy (52)<br>- Justice as fairness (53)		49
2	Reciprocity (2, 4, 6, 8- 9, 12, 14, 16, 19, 31, 33, 35, 39-40, 42, 54-62)	- Mutual exchange (12)<br>- Society and employers should support and protect those who take on increased burden and risk (2, 4, 9, 39)<br>- Support for those enduring a disproportionate burden during crisis and address/minimize burden (42)<br>- Obligations to healthcare workers (14)<br>- Justice-orientated reciprocity (62)	- Prioritisation of care (55)<br>- Openness (12)<br>- Duty to safeguard, duty to guide, duty to plan (54)<br>- Limits of responsibilities (61)<br>- Ethical duty to protect employees (54)<br>- Risk to employees (e.g. healthcare employees) should be minimised (6)<br>- Employers have a duty to their employees (58)<br>- "*If people are asked to take increased risks, or face increased burdens during a pandemic influenza, they should be supported in doing so, and the risks and burdens should be minimized as far as possible*" (12)<br>- "*Reciprocity requires that society supports those who face a disproportionate burden in protecting the public good and takes steps to minimize their impact as far as possible." Additional consideration is required to determine how best to put this principle into practice*" (33)	24

3	Transparency (2, 9, 15, 28, 30, 36-37, 42, 44, 49, 56, 59, 63-69)	- Openness and public accessibility (30, 42)<br>- Communication (33, 56)<br>- Publicly defensible (42)<br>- Justification (49)<br>- Veracity (70)	- Accountability (44, 49, 65)<br>- Obligations to community (30)<br>- Trust (44)<br>- Monitoring and evaluation (65)	21
4	(Social) (Distributive) Justice (8, 13, 17- 18, 28, 34, 44-45, 49, 51, 57, 63-64, 70-73)	- Justice as fairness (53)		18
5	Duty to (provide) care (4, 16, 19, 22, 34, 40, 42, 55, 59, 63-64, 66, 74- 75)	- Professional obligation of non-abandonment (19)<br>- Professional duty to respond (42)<br>- Professional responsibility (44)<br>- Duty to treat (76)<br>- The obligation of healthcare workers to serve under stressful and risky conditions (77)<br>- Professional integrity (78)	- *"Protect heath workers in the midst of the crisis"* (10)<br>- Risk life to satisfy duty (22)<br>- Willingness to work (22)<br>- Conflicting professional duties (32)<br>- Health worker motivation (78)	18
6	Individual Liberty (8, 19, 27, 34- 35, 42, 55, 60, 79- 80)	- Liberty (33, 49, 64, 74)<br>- Least restrictive (10, 22, 42)<br>- Autonomy (19, 80)<br>- Constraints on / restrictions of liberty (27, 34- 35)<br>- Individual autonomy (15)<br>- Equal liberty and human rights (44)<br>- Patient autonomy (79)<br>- Patient liberty (60)<br>- Choice, Free-will, Self-determination (55)	- Balancing public health and civil liberties (10)<br>- *"Limitations to rights of individual during public health crisis. Proportionality, protecting public good, least restrictive means, without discrimination to certain groups"* (42)	18
7	Utility (3, 11, 20, 26, 28, 39, 55, 59, 81)	- Efficiency (39)<br>- Effectiveness (55)<br>- Greatest good for the greatest number (55)<br>- Utilitarian value (82)	- When resources are scarce, it may be necessary to allocate them where they will provide the greatest impact on society (55)	10
8	Stewardship (11, 17, 25, 36, 40, 42, 46, 64, 66, 83- 84)	- Governance (15)<br>- Duty to steward resources (63)	- "*Duty to ensure that vital resources are employed efficiently*" (17)<br>- Good outcomes (benefits to the public good). (42)	13

			- Equity (fair distribution of benefits and burdens) (42)<br>- Protect and develop resources (42)<br>- *“Clinicians will need to balance the obligation to save the greatest possible number of lives against that of the obligation to care for each single patient”* (64)<br>- *“Wise decisions must be while allocating scarce resources with the 'goal of maximising good while minimizing harm'”* (79)<br>- Distributive justice (84)	
9	Trust (16, 31- 32, 42, 59, 68-69, 74, 85)	- Informed and trusted communication (10)<br>- Fidelity (70)<br>- Honouring Patients’ Trust (78)	- Transparency (59, 68- 69)<br>- Trust between the patient and clinician / the care team (42, 70), between staff the employers, between organisations and between the public and health care providers (42)<br>- *“Maintaining trust in tension with having to impose limits”* (42)<br>- Ethical processes stewardship (42)<br>- Trust from society (68)<br>- Continued education of the public is necessary (69)<br>- Community consent and accountability (85)<br>- Triage policies (85)	12
10	Proportionality (12, 15- 16, 29, 41- 42, 46, 68, 80)	- Fair procedures (22)	- *“The compromise that individuals have to make for the sake of common good should be proportional to the benefits”* (15)<br>- Balancing personal autonomy and community well-being/benefit (30)<br>- *“Personal liberty/rights- in tension with restrictions. Restrictions to individual/group in proportion to risk to public health Justifies use of more coercive measures when least coercive measure have failed to achieve appropriate “ends””*(42)	10

11	Beneficence (44, 70- 71, 86)	- Avoid harm, harm reduction, minimising harm (2, 9, 10, 44)<br>- Nonmaleficence (86)		9
12	Accountability (15, 30, 33, 42, 44, 49, 63, 66)		- Transparency (44, 49)<br>- Liability (23)<br>- Good preparedness practice (30)<br>- Reasonableness (33)<br>- Justification (49)<br>- Duty to plan (63)	8
13	Protection of the Public from Harm (10, 16, 31, 42)	- Good preparedness (30)<br>- Protection of individuals at highest risk, meeting societal needs, and promoting social justice (10)<br>- Ensuring that benefits of relief and rescue activities reach the affected (15)	- Avoiding, preventing and removing harms in the community (15)<br>- *"Public well-being & safety- in tension with individual autonomy. Compliance of individuals for public good. Rationing –priority setting. Least restrictive means used. Transparency of consequences Individual's interest in the well-being of community"* (42)	6
14	Privacy (4, 30, 42, 49, 60)		- Good preparedness practice (30)<br>- *"Right to privacy in tension with demands of crisis for shared information. Proportionality Protection from stigmatization. Disclose only that which is necessary to protect public health"* (42)	5
15	Autonomy (45, 67, 70, 86)		- *"Patient autonomy involves a number of different concepts, including privacy, voluntariness, self-mastery, free choice, choosing one's own moral position, and accepting responsibility for one's choices. These concepts define a "respect for autonomy" that is summarized as "personal rule of self by adequate understanding while remaining free from controlling influences by others and from personal limitations that prevent choice." Respect for a person's autonomy covers their thoughts, will, and actions"* (70)	4
16	Solidarity (5, 42, 87)	- Mutual responsibility (56)		4

17	Community participation (15, 30)	- Community resilience and empowerment (44)<br>- Obligations to community (30)		4
18	Working together (2, 9, 12)		-Helping one another. (12)<br>- *"Being prepared to share information that will help others, without compromising the privacy and dignity of the individuals involved. Because pandemic influenza will affect the whole of society, it is important that different agencies collaborate and coordinate at provincial, regional and local levels. Working together also implies strong links at the international, national and inter-provincial levels. This includes both providing and seeking timely information from partners"* (12)<br>- "*We need to support each other, take responsibility for our own behaviour and share information appropriately*" (2)	3
19	Responsiveness (15, 42)	- Responsiveness to local values (88)		3
20	Consistency (63, 66)			2
21	Duty to Plan (63)	- Flexibility and adaptability (2)	- Duty to plan and accountability (63)	2
22	Evidence (30)		- Standards (30)<br>- Guidelines (30)<br>- Good preparedness practice (30)	1
23	Others: related to Social-Community	- Respect (2, 25)<br>- Social cohesiveness and collaboration (24)<br>- Responsive civic response (44)<br>- Dignity (56)<br>- Compassion (89)		
24	Others: related to decision-making processes	- Reasonableness (42)<br>- Inclusiveness (42)<br>- Sustainability (sustainable action and sustainable outcomes) (15)<br>- Relevance (15)		

REFERENCES

1. Chisholm J. Doctors will have to choose who gets life-saving treatment. Here's how we'll do it | John Chisholm. The Guardian [Internet]. 2020; Available from: https://www.theguardian.com/commentisfree/2020/apr/01/doctors-choose-life-saving-treatment-ethical-rules

2. BMA. COVID-19: ethical issues. Br Med Assoc Trade Union Prof Body Dr UK [Internet]. 2020; Available from: https://www.bma.org.uk/advice-and-support/covid-19/ethics/covid-19-ethical-issues

3. Emanuel EJ, Persad G, Upshur R, Thome B, Parker M, Glickman A, et al. Fair Allocation of Scarce Medical Resources in the Time of Covid-19. N Engl J Med [Internet]. 2020; Available from: https://doi.org/10.1056/NEJMsb2005114

4. Department of Health , Ireland. Ethical framework for decision-making in a pandemic [Internet]. 2020 [cited 2021 Feb 4]. Available from: https://www.gov.ie/en/publication/dbf3fb-ethical-framework-for-decision-making-in-a-pandemic/

5. Johns Hopkins Berman Institute of Bioethics. Overview: Ethical Concerns in Responding to Coronavirus [Internet]. Johns Hopkins Berman Institute of Bioethics. 2020. Available from: https://bioethics.jhu.edu/news-events/news/coronavirus-ethical-concerns-in-planning-a-response/

6. MSCBS. Informe del Ministerio de Sanidad sobre los aspectos éticos en situaciones de pandemia: El SARS-CoV-2. 2020; Available from: https://www.mscbs.gob.es/profesionales/saludPublica/ccayes/alertasActual/nCov-China/documentos/AspectosEticos_en_situaciones_de_pandemia.pdf

7. Rosenbaum L. Facing Covid-19 in Italy — Ethics, Logistics, and Therapeutics on the Epidemic's Front Line. N Engl J Med [Internet]. 2020; Available from: https://www.nejm.org/doi/10.1056/NEJMp2005492

8. White DB, Lo B. A Framework for Rationing Ventilators and Critical Care Beds During the COVID-19 Pandemic. JAMA [Internet]. 2020; Available from: https://jamanetwork.com/journals/jama/fullarticle/2763953

9. Scottish Government. Coronavirus (COVID-19): ethical advice and support framework. 2020; Available from: https://www.gov.scot/publications/coronavirus-covid-19-ethical-advice-and-support-framework/

10. Gostin. Responding to COVID-19: How to Navigate a Public Health Emergency Legally and Ethically. 2020; Available from: https://onlinelibrary.wiley.com/doi/full/10.1002/hast.1090

11. Ram-Tiktin E. Ethical Considerations of Triage Following Natural Disasters: The IDF Experience in Haiti as a Case Study. Bioethics. 2017;31(6):467–75.

12. Alberta Government. Alberta's Ethical Framework for Responding to Pandemic Influenza. 2016;15.

13. Krütli P, Rosemann T, Törnblom KY, Smieszek T. How to Fairly Allocate Scarce Medical Resources: Ethical Argumentation under Scrutiny by Health Professionals and Lay People. PloS One. 2016;11(7):e0159086.

14. Lor A, Thomas JC, Barrett DH, Ortmann LW, Herrera Guibert DJ. Key Ethical Issues Discussed at CDC-Sponsored International, Regional Meetings to Explore Cultural Perspectives and Contexts on Pandemic Influenza Preparedness and Response. Int J Health Policy Manag. 2016;5(11):653–62.

15. Mariaselvam S, Gopichandran V. The Chennai floods of 2015: urgent need for ethical disaster management guidelines. Indian J Med Ethics. 2016;1(2):91–5.

16. Downar J., Seccareccia D. Palliating a Pandemic: 'All Patients Must Be Cared For'. J Pain Symptom Manage. 2010;39(2):291–5.

17. Kipnis K. Overwhelming casualties: medical ethics in a time of terror. Account Res. 2003;10(1):57–68.

18. McLachlan HV. A proposed non-consequentialist policy for the ethical distribution of scarce vaccination in the face of an influenza pandemic. J Med Ethics. 2012;38(5):317–8.

19. McLean MM. Ethical Preparedness for Pandemic Influenza: A Toolkit. 2012; Available from: https://www.scu.edu/ethics/focus-areas/bioethics/resources/ethical-preparedness-for-pandemic-influenza/

20. Rubenfeld GD. Cost-effective critical care: cost containment and rationing. Semin Respir Crit Care Med. 2012;33(4):413–20.

21. Antommaria AHM, Powell T, Miller JE, Christian MD, Task Force for Pediatric Emergency Mass Critical Care. Ethical issues in pediatric emergency mass critical care. Pediatr Crit Care Med J Soc Crit Care Med World Fed Pediatr Intensive Crit Care Soc. 2011 Nov;12(6 Suppl):S163-168.

22. Devnani M, Gupta AK, Devnani B. Planning and response to the influenza A (H1N1) pandemic: ethics, equity and justice. Indian J Med Ethics. 2011;8(4):237–40.

23. Koenig K.L., Lim H.C.S., Tsai S.-H. Crisis Standard of Care: Refocusing Health Care Goals During Catastrophic Disasters and Emergencies. J Exp Clin Med. 2011;3(4):159–65.

24. Vawter D.E., Gervais K.G., Garrett J.E. Allocating pandemic influenza vaccines in Minnesota: Recommendations of the Pandemic Influenza Ethics Work Group. Vaccine. 2007;25(35):6522–36.

25. Tillyard A. Reorganising the pandemic triage processes to ethically maximise individuals' best interests. Intensive Care Med. 2010;36(11):1966–71.

26. Silva DS, Nie JX, Rossiter K, Sahni S, Upshur REG, Canadian Program of Research on Ethics in a Pandemic. Contextualizing ethics: ventilators, H1N1 and marginalized populations. Healthc Q Tor Ont. 2010;13(1):32–6.

27. Consortium of Missouri Health Ethics Organizations. Health Ethics Considerations: Planning for and Responding to Pandemic Influenza in Missouri. 2009;

28. Hoffman M. Editorial comments-rationing of resources: ethical issues in disasters and epidemic situations. Prehospital Disaster Med. 2009;24(3):222.

29. Institute of Medicine (US) Committee on Guidance for Establishing Standards of Care for Use in Disaster Situations, Altevogt BM, Stroud C, Hanson SL, Hanfling D, Gostin LO. Guidance for Establishing Crisis Standards of Care for Use in Disaster Situations: A Letter Report [Internet]. Washington (DC): National Academies Press (US); 2009. Available from: http://www.ncbi.nlm.nih.gov/books/NBK219958/

30. Barnett DJ, Taylor HA, Hodge JG, Links JM. Resource allocation on the frontlines of public health preparedness and response: report of a summit on legal and ethical issues. Public Health Rep Wash DC 1974. 2009;124(2):295–303.

31. Frolic A, Kata A, Kraus P. Development of a critical care triage protocol for pandemic influenza: integrating ethics, evidence and effectiveness. Healthc Q Tor Ont. 2009;12(4):54–62.

32. Lauridsen S. Administrative gatekeeping - a third way between unrestricted patient advocacy and bedside rationing. Bioethics. 2009;23(5):311–20.

33. Levin D, Cadigan RO, Biddinger PD, Condon S, Koh HK, Joint Massachusetts Department of Public Health-Harvard Altered Standards of Care Working Group. Altered standards of care during an influenza pandemic: identifying ethical, legal, and practical principles to guide decision making. Disaster Med Public Health Prep. 2009;3:S132-140.

34. Pena ME, Irvin CB, Takla RB. Ethical considerations for emergency care providers during pandemic influenza--ready or not.. Prehospital Disaster Med. 2009;24(2):115–9; discussion 120.

35. Verweij M. Moral principles for allocating scarce medical resources in an influenza pandemic. J Bioethical Inq. 2009;6(2):159–69.

36. Bostick NA, Subbarao I, Burkle FM, Hsu EB, Armstrong JH, James JJ. Disaster triage systems for large-scale catastrophic events. Disaster Med Public Health Prep. 2008;2:S35-39.

37. Devereaux AV, Dichter JR, Christian MD, Dubler NN, Sandrock CE, Hick JL, et al. Definitive care for the critically ill during a disaster: a framework for allocation of scarce resources in mass critical care: from a Task Force for Mass Critical Care summit meeting, January 26-27, 2007, Chicago, IL. Chest. 2008;133(5):51S-66S.

38. Tabery J, Mackett CW, University of Pittsburgh Medical Center Pandemic Influenza Task Force's Triage Review Board. Ethics of triage in the event of an influenza pandemic. Disaster Med Public Health Prep. 2008;2(2):114–8.

39. WHO. Addressing ethical issues in pandemic influenza planning DISCUSSION PAPERS. 2008;

40. Daugherty Biddison L., Berkowitz K.A., Courtney B., De Jong M.J., Devereaux A.V., Kissoon N., et al. Ethical considerations: Care of the critically ill and injured during pandemics and disasters: CHEST consensus statement. Chest. 2014;146:e145S-e155S.

41. Christian M.D., Hawryluck L., Wax R.S., Cook T., Lazar N.M., Herridge M.S., et al. Development of a triage protocol for critical care during an influenza pandemic. CMAJ. 2006;175(11):1377–81.

42. Thompson AK, Faith K, Gibson JL, Upshur REG. Pandemic influenza preparedness: an ethical framework to guide decision-making. BMC Med Ethics. 2006;7:E12.

43. Melnychuk RM, Kenny NP. Pandemic triage: the ethical challenge. CMAJ Can Med Assoc J J Assoc Medicale Can. 2006;175(11):1393–4.

44. CDC. CDC - ACD Ethics Subcommittee Documents - OSI - OS. 2019; Available from: https://www.cdc.gov/os/integrity/phethics/ESdocuments.htm

45. Kukora S., Laventhal N. Choosing wisely: should past medical decisions impact the allocation of scarce ECMO resources? Acta Paediatr Int J Paediatr. 2016;105(8):876–8.

46. Hick JL, DeVries AS, Fink-Kocken P, Braun JE, Marchetti J. Allocating resources during a crisis: you can't always get what you want. Minn Med. 2012;95(4):46–50.

47. Caro JJ, Coleman CN, Knebel A, DeRenzo EG. Unaltered ethical standards for individual physicians in the face of drastically reduced resources resulting from an improvised nuclear device event. J Clin Ethics. 2011;22(1):33–41.

48. Knebel AR, Coleman CN, Cliffer KD, Murrain-Hill P, McNally R, Oancea V, et al. Allocation of scarce resources after a nuclear detonation: setting the context. Disaster Med Public Health Prep. 2011;5:S20-31.

49. Kass NE. An ethics framework for public health and avian influenza pandemic preparedness. Yale J Biol Med. 2005;78(5):239–54.

50. Wolf L, Hensel W. Valuing lives: Allocating scarce medical resources during a public health emergency and the Americans with Disabilities Act (perspective). PLoS Curr. 2011;3:RRN1271.

51. Rosoff PM, DeCamp M. Preparing for an influenza pandemic: are some people more equal than others? J Health Care Poor Underserved. 2011;22(3):19–35.

52. Persad G, Wertheimer A, Emanuel EJ. Principles for allocation of scarce medical interventions. Lancet Lond Engl. 2009;373(9661):423–31.

53. Roberts MJ, Reich MR. Ethical analysis in public health. The Lancet. 2002;359(9311):1055–9.

54. Berlinger N, Wynia M, Powell T. Ethical Framework for Health Care Institutions & Guidelines for Institutional Ethics Services Responding to the Coronavirus Pandemic. Hastings Cent [Internet]. 2020; Available from: https://www.thehastingscenter.org/ethicalframeworkcovid19/

55. Moodley K, Hardie K, Selgelid MJ, Waldman RJ, Strebel P, Rees H, et al. Ethical considerations for vaccination programmes in acute humanitarian emergencies. Bull World Health Organ. 2013;91(4):290–7.

56. Silva DS, Gibson JL, Robertson A, Bensimon CM, Sahni S, Maunula L, et al. Priority setting of ICU resources in an influenza pandemic: a qualitative study of the Canadian public's perspectives. BMC Public Health. 2012;12:241.

57. Rothstein MA. Currents in contemporary ethics. Should health care providers get treatment priority in an influenza pandemic? J Law Med Ethics J Am Soc Law Med Ethics. 2010;38(2):412–9.

58. Simonds AK, Sokol DK. Lives on the line? Ethics and practicalities of duty of care in pandemics and disasters. Eur Respir J. 2009;34(2):303–9.

59. Garrett JE, Vawter DE, Prehn AW, DeBruin DA, Gervais KG. Ethical considerations in pandemic influenza planning. Minn Med. 2008;91(4):37–9.

60. Holt GR. Making difficult ethical decisions in patient care during natural disasters and other mass casualty events. Otolaryngol--Head Neck Surg Off J Am Acad Otolaryngol-Head Neck Surg. 2008;139(2):181–6.

61. Wynia MK. Ethics and public health emergencies: encouraging responsibility. Am J Bioeth AJOB. 2007;7(4):1–4.

62. Strous R.D., Gold A. Ethical lessons learned and to be learned from mass casualty events by terrorism. Curr Opin Anaesthesiol. 2019;32(2):174–8.

63. Ryus C, Baruch J. The Duty of Mind: Ethical Capacity in a Time of Crisis. Disaster Med Public Health Prep. 2018;12(5):657–62.

64. Zucker HA, Adler KP, Berens DP. Ventilator Allocation Guidelines. New York State Task Force on Life and the Law New York State Department of Health. 2015;272.

65. Gostin LO. Ethical allocation of drugs and vaccines in the West African ebola epidemic. Milbank Q. 2014;92(4):662–6.

66. Hick JL, Hanfling D, Cantrill SV. Allocating scarce resources in disasters: emergency department principles. Ann Emerg Med. 2012;59(3):177–87.

67. Schuklenk U, Gartland KMA. Confronting an influenza pandemic: ethical and scientific issues. Biochem Soc Trans. 2006;34:1151–4.

68. Kinlaw K, Barrett DH, Levine RJ. Ethical guidelines in pandemic influenza: recommendations of the Ethics Subcommittee of the Advisory Committee of the Director, Centers for Disease Control and Prevention. Disaster Med Public Health Prep. 2009;3:S185-192.

69. O'Laughlin DT, Hick JL. Ethical issues in resource triage. Respir Care. 2008;53(2):190–7; discussion 197-200.

70. Repine T.B., Lisagor P., Cohen D.J. The dynamics and ethics of triage: Rationing care in hard times. Mil Med. 2005;170(6):505–9.

71. Lin JY, Anderson-Shaw L. Rationing of resources: ethical issues in disasters and epidemic situations. Prehospital Disaster Med. 2009;24(3):215–21.

72. Fortes PA de C, Zoboli ELCP. A study on the ethics of microallocation of scarce resources in health care. J Med Ethics. 2002;28(4):266–9.

73. Hick JL, Rubinson L, O'Laughlin DT, Farmer JC. Clinical review: allocating ventilators during large-scale disasters--problems, planning, and process. Crit Care Lond Engl. 2007;11(3):217.

74. Lo B, Katz MH. Clinical decision making during public health emergencies: ethical considerations. Ann Intern Med. 2005;143(7):493–8.

75. Leider JP, DeBruin D, Reynolds N, Koch A, Seaberg J. Ethical Guidance for Disaster Response, Specifically Around Crisis Standards of Care: A Systematic Review. Am J Public Health. 2017;107(9):e1–9.

76. Bailey TM, Rosychuk RJ, Yonge O, Marrie TJ. A Duty to Treat During a Pandemic? The Time for Talk is Now. Am J Bioeth [Internet]. 2008; Available from: http://www.tandfonline.com/doi/abs/10.1080/15265160802318220

77. Bhatia P. The H1N1 influenza pandemic: need for solutions to ethical problems. Indian J Med Ethics. 2013;10(4):259–63.

78. Eyal N., Firth P. Repeat triage in disaster relief: Questions from Haiti. PLoS Curr [Internet]. 2012; Available from: http://www.embase.com/search/results?subaction=viewrecord&from=export&id=L366284225

79. Good L. Ethical decision making in disaster triage. J Emerg Nurs. 2008;34(2):112–5.

80. Schröder P, Brand H, Schröter M, Brand A. [Ethical discussion on criteria for policy makers in public health authorities for preventative measures against a pandemic caused by a novel influenza A virus]. Gesundheitswesen Bundesverb Arzte Offentlichen Gesundheitsdienstes Ger. 2007;69(6):371–6.

81. Wagner JM, Dahnke MD. Nursing Ethics and Disaster Triage: Applying Utilitarian Ethical Theory. J Emerg Nurs. 2015;41(4):300–6.

82. Jecker NS, Dudzinski DM, Diekema DS, Tonelli M. Ebola Virus Disease: Ethics and Emergency Medical Response Policy. Chest. 2015;148(3):794–800.

83. Kass NE, Otto J, O'Brien D, Minson M. Ethics and severe pandemic influenza: maintaining essential functions through a fair and considered response. Biosecurity Bioterrorism Biodefense Strategy Pract Sci. 2008;6(3):227–36.

84. Batter P.M. Influenza pandemic planning for cancer patients. Curr Oncol [Internet]. 2006;13(4). Available from: http://www.embase.com/search/results?subaction=viewrecord&from=export&id=L352335890

85. Kraus CK, Levy F, Kelen GD. Lifeboat ethics: considerations in the discharge of inpatients for the creation of hospital surge capacity. Disaster Med Public Health Prep. 2007;1(1):51–6.

86. Kirby J. Enhancing the fairness of pandemic critical care triage. J Med Ethics. 2010;36(12):758–61.

87. Kuschner WG, Pollard JB, Ezeji-Okoye SC. Ethical triage and scarce resource allocation during public health emergencies: tenets and procedures. Hosp Top. 2007;85(3):16–25.

88. Trotter G. Sufficiency of care in disasters: ventilation, ventilator triage, and the misconception of guideline-driven treatment. J Clin Ethics. 2010;21(4):294–307.

89. Bell JAH, Hyland S, DePellegrin T, Upshur REG, Bernstein M, Martin DK. SARS and hospital priority setting: a qualitative case study and evaluation. BMC Health Serv Res. 2004;4(1):36.
